# Childhood transgenderity under the perspective of elementary school teachers*

**DOI:** 10.1590/1518-8345.3792.3459

**Published:** 2021-06-28

**Authors:** Francisca Vilena da Silva, Renata Dantas Jales, Ivoneide Lucena Pereira, Luana Rodrigues de Almeida, Jordana de Almeida Nogueira, Sandra Aparecida de Almeida

**Affiliations:** 1 Universidade Federal da Paraíba, João Pessoa, PB, Brazil.; 3 Universidade Federal da Paraíba, Centro de Ciências da Saúde, João Pessoa, PB, Brazil.

**Keywords:** Transgender Persons, Gender Dysphoria, Gender Identity, Preschool Child, Child Rearing, Faculty, Pessoas Transgênero, Disforia de Gênero, Identidade de Gênero, Pré-Escolar, Educação Infantil, Docentes, Pessoas Transgênero, Disforia de Género, Identidad de Género, Preescolar, Crianza del Niño, Docentes

## Abstract

**Objective:**

to analyze teachers’ conceptions about transgenderity in childhood and to identify the possibilities and limits of working with these children in the school context.

**Method:**

a qualitative research study, carried out with 23 teachers from two municipal elementary schools. Semi-structured interviews were used to produce empirical material. As an analytical resource, the content analysis technique, thematic modality, was used.

**Results:**

six thematic categories emerged in the set of empirical material: *There is transgenderity in childhood; The construction of gender identity and roles in childhood; The experience of trans children in the school context; Trans children: How to deal with?; Discussing the differences in the classroom: Is this the way?; and Dilemmas of school and family interaction*. It was found that the gender dichotomy is reinforced in the classroom, causing tensions and stereotyped divisions for male and female roles. Various forms of violence have been reproduced by classmates and teachers, who, due to lack of knowledge or to unpreparedness, reinforce concepts and attitudes that lead to the maintenance of exclusion.

**Conclusion:**

the schools find it difficult to promote the inclusion of trans children. It is necessary to create strategies aimed at raising awareness and training the professionals who make up the school environment, especially teachers in the initial grades.

## Abstract (pt)

**Objetivo:**

analisar as concepções de professores sobre a transgeneridade na infância e identificar as possibilidades e os limites de trabalho com essas crianças no contexto escolar.

**Método:**

pesquisa qualitativa, realizada com 23 professores de duas escolas municipais de ensino fundamental. Para produção do material empírico, empregaram-se entrevistas semiestruturadas. Como recurso analítico recorreu-se à técnica de análise de conteúdo, modalidade temática.

**Resultados:**

no conjunto do material empírico, emergiram seis categorias temáticas: *Transgeneridade na infância existe; A construção de identidade e papéis de gênero na infância; A vivência de crianças trans no contexto escolar; Crianças trans: como lidar?; Discutir as diferenças em sala de aula: é o caminho?; Dilemas da interação escola e família*. Constatou-se que a dicotomia de gênero é reforçada na sala de aula, ocasionando tensões e divisões estereotipadas para os papéis masculino e feminino. Diversas formas de violência vêm sendo reproduzidas por coleguinhas e professores, que por falta de conhecimento ou despreparo reforçam concepções e atitudes que levam à manutenção da exclusão.

**Conclusão:**

as escolas têm dificuldades para promover a inclusão de crianças trans. Faz-se necessária a criação de estratégias voltadas para a sensibilização e capacitação dos profissionais que compõem o ambiente escolar, sobretudo os professores das séries iniciais.

## Abstract (es)

**Objetivo:**

analizar las concepciones de los docentes sobre la transgeneridad en la infancia e identificar las posibilidades y límites de trabajar con estos niños en el contexto escolar.

**Método:**

investigación cualitativa, realizada con 23 profesores de dos escuelas primarias municipales. Para la producción de material empírico se utilizaron entrevistas semiestructuradas. Como recurso analítico se utilizó la técnica de análisis de contenido, modalidad temática.

**Resultados:**

de todo el material empírico surgieron seis categorías temáticas: *La transgeneridad en la infancia existe; Construcción de roles de identidad y género en la infancia; Experiencia de los niños trans en el contexto escolar; Niños trans: ¿cómo tratarlos? Discutir las diferencias en el aula: ¿es el camino?; Dilemas de la interacción escuela y familia*. Se verificó que la dicotomía de género se refuerza en el aula, provocando tensiones y divisiones estereotipadas para los roles masculino y femenino. Compañeros y profesores vienen reproduciendo diversas formas de violencia, por desconocimiento o falta de preparación refuerzan conceptos y actitudes que sostienen la exclusión.

**Conclusión:**

las escuelas tienen dificultades para promover la inclusión de niños trans. Es necesario crear estrategias dirigidas a sensibilizar y capacitar a los profesionales que integran el entorno escolar, especialmente a los docentes en los grados iniciales.

## Introduction

Early in the beginning of life, children understand the discourses about what it is to be a boy or a girl and what is allowed to each one. From an early age, framing a particular gender is permeated by social and family influences, outlining behaviors, tastes and feelings based on a heteronormative conception^(^ 1 ^-^ 2 ^)^. This process favors/reinforces the matrix of binarism, developing gender roles in subjects from an early age according to situational conditions, with their biological bodies and pleasures^(^ 3 ^)^.

In today’s society, gender, as a concept, provides for male/female binarism and refers to ways of identifying oneself and being identified as male or female, and this, as far as it is concerned, is transversal to cultural, ethnic, racial, political and economic aspects. In its turn, sex represents the male/female binary, the one already determined at birth according to the child’s genitals. The truth is that the choice to which pole the individual should belong is something socially pre-established even before the child comes into the world^(^ 4 ^-^ 5 ^)^.

Understanding reality through a dichotomous view is one of the premises of maintaining binarism that encircles the constitution of singularities. Concepts such as gender binary and heteronormativity dictate patterns and regulate bodies, with plastered concepts that do not account for the subjects’ complexity and diversity. Such circumstances promote discrimination and produce marks on those who dare to break or modify the hegemonic norms and pedagogies^(^ 2 ^,^ 5 ^)^. Furthermore, they influence the maintenance of a culture of exclusion, especially those who do not fully assume the behavior and/or roles considered to belong to the gender corresponding to birth sex^(^ 6 ^)^.

Sometimes, the non-alignment with the sex/gender system can result in the search for bodily and behavioral adaptations converging with the gender with which the person is identified: this is called transgenderity^(^ 4 ^)^. Transgenderity (trans) is a possible condition for individuals to assume a gender identity, different from that designated at the time of their birth^(^ 2 ^)^. In other words, it describes a person whose gender identity is incongruous (or does not “coincide”) with the biological sex^(^ 6 ^)^.

When it comes to childhood transgenderity, it is necessary to highlight that it is a controversial topic and requires greater dissemination^(^ 7 ^-^ 8 ^)^. Although the scientific production is restricted, a number of studies show that the family universe is an initial space for socialization, where the first affective and sexual experiences occur^(^ 7 ^-^ 9 ^)^. The ways in which a family adjusts and is affected by the child’s trans identity can compromise their development and access to resources related to their gender identity. Experiences of family silencing, imposition of heteronormativity, discrimination, hostility and non-acceptance of the condition of gender identity negatively interfere in the process of social inclusion^(^ 10 ^)^.

Nevertheless, movements of resistance and exclusion are recurrent and cross the discourses of the school environment. While recognizing the social role of school institutions in making a commitment to diversity and minimizing forms of prejudice, whether ethnic-racial, gender and/or sexual orientation, such structures and subjects involved in them still try to put out those who resist normalization of their sexual and gender identities based on hegemonic standards^(^ 11 ^)^.

In view of this, school dropout in these individuals has become more frequent as they suffer insults and aggressions from students and teachers who imbue with them the justification that these people have an appearance and behavior not suitable for their biological gender. The impossibility of using the social name, heteronormative discipline, constraints for using the restroom, bullying and segregation stand out^(^ 8 ^)^. Such issues have become problematic for the lives of those considered socially different, being exposed to situations that may come to lead to mental illness, with increased risk for suicide, stress and anxiety^(^ 12 ^-^ 14 ^)^, in addition to being prevented from enjoying their rights as citizens^(^ 15 ^)^.

A research study carried out in Norway with higher education students showed that transgender (trans) people had a significantly higher number of psychosocial demands, related to loneliness, mental health problems, self-esteem and suicidal behavior, when compared to individuals whose gender identities or expressions are congruent with the sex attributed at birth^(^ 16 ^)^. Additionally, for illustrative purposes, a study conducted with medical students from the Boston University-USA showed that the self-reported knowledge on transgender’s health is deficient, requiring targeted training for this population^(^ 17 ^)^.

The processes of maintaining institutional invisibility and the supposedly unnatural nature of childhood transgenderity have actively contributed to the unpreparedness, fear and inability of educators to deal with this demand^(^ 18 ^)^. Without preparation and clarification, the learning space does not welcome, recognize or encourage trans and/or non-binary children, leaving them with stigmatization and discrimination^(^ 19 ^)^.

This scenario leads to reflection on the importance of unveiling aspects that are still obscure about childhood transgenderity in the school context. It is assumed that elementary school teachers have an impaired understanding of the theme and thus contribute to the school becoming a space for exclusion and spread of violence.

In view of the above, the study aims to analyze teachers’ conceptions about transgenderity in childhood and to identify the possibilities and limits of working with these children in the school context.

## Method

A descriptive study with qualitative data analysis^(^ 20 ^)^, conducted in two municipal elementary schools in the city of João Pessoa/PB. The schools were appointed by the manager of the Municipal Secretariat of Education, considering the following specificities as selection criteria: distinct pedagogical approaches, one with a traditional educational system, more restricted to pre-established content in textbooks and manuals (School A) and the other, responsible for more open and comprehensive education, in order to meet the students’ needs (School B).

**Figure 1:**
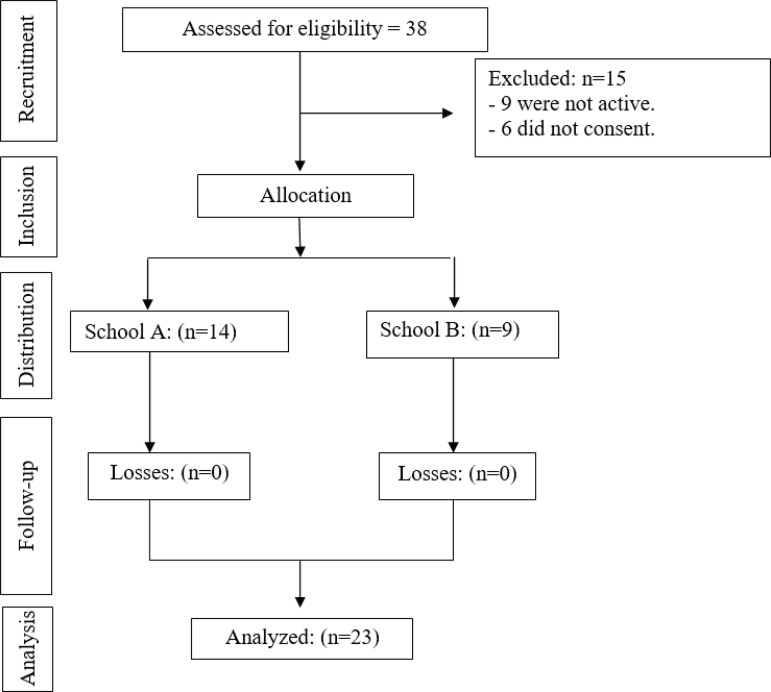
Research flowchart. João Pessoa, Brazil, 2019

In order to recruit potential participants, there was previous assistance of the managers from both schools, who passed on information on the total number of teachers, name, grade in which they taught, length of service, and whether they were working at the time of the research. In the initial survey, 38 elementary school teachers registered in the two Schools (A and B) were identified. Of these, nine were away from their duties (vacation, leave), and were not included in the survey. Subsequently, a meeting was held in each educational establishment, in which the objectives, the importance of the research, its risks and benefits were passed on to the teachers. Of the total of 29 teachers, six refused to participate in the research due to religious and personal issues. Thus, 23 teachers were included, 14 from School A and nine from School B (Figure 1).

In order to allow for a better understanding of the study design, in this research, the Consolidated Criteria for Reporting Qualitative Research (COREQ) were considered as a support tool^(^ 21 ^)^. The semi-structured interview technique was employed, using a script that addressed issues related to the professionals’ knowledge about transgenderity, their experiences with the theme, their limits and suggestions for working on the theme in the classroom. The interviews lasted a mean of 40 minutes, were recorded and conducted by the first author of the study. In order to preserve privacy and minimize any discomfort, the interviews were conducted in a private and reserved place in the schools. The recorded interviews were transcribed by two individuals shortly after their completion, being checked by a third party, in order to maintain scientific rigor.

To analyze the collected data, the Content Analysis technique was used in its thematic modality, which occurred at three moments: 1) pre-analysis: the synthesis of the initial ideas for the interpretation of the collected information was carried out, according to the principles of completeness, representativeness, homogeneity and pertinence; 2) exploration of the material: the excerpts were coded according to the repetition of the words, building up the registration units from which the thematic categories were derived. Initially, twenty initial categories originated: from those, eleven intermediate categories emerged, through which the six final categories of the study were constituted: *There is transgenderity in childhood; The construction of gender identity and roles in childhood; The experience of trans children in the school context; Trans children: How to deal with?; Discussing the differences in the classroom: Is this the way?; and Dilemmas of school and family interaction*; and finally, the treatment, inference and interpretation of the results were performed^(^ 22 ^)^.

The ethical criteria were respected, and the study was approved under protocol No. 2,983,380/CAEE 95992318.6.0000.5188. Each interviewee participated voluntarily in the research and signed the Free and Informed Consent Form. To guarantee the confidentiality of the information and the anonymity of the participants, a sequential number was assigned to the interviews (P.1, P.2, P.3.....P.23), followed by the Schools identification code (E.A and E.B).

## Results

Regarding the characterization of the participating teachers, there was predominance of females in both schools. The median age was 50 years old at School A, and 35 years old at School B. There was predominance of married teachers at School A, who declared themselves Evangelical or Catholic. At School B, single individuals prevailed, with no defined religion. Previous training to work on issues involving sexual and gender diversity was incipient, although relatively superior at School B.

The first category, “*There is transgenderity in childhood*”, showed elements related to the existence or not of transgender children. Different positions were identified among the teachers, going through genetic and scientific conceptions or as a non-existent reality. The understanding that genetic factors are associated with transgenderity was highlighted in the following statement: *It is something that already comes into the person’s genes, that the child is born with that, was born a girl and was born with another sex, but that already comes in it (P.01, E.A)*.

For other teachers, childhood transgenderity is characterized from the view of a body that does not belong to their gender identity, in which boys and girls, from the moment they become aware of their gender role imposed by society, are not identify with it. *I believe that transgenderity occurs in childhood from the moment they have one body and see themselves in another, it changes based on the desires if she is a girl the desire of being a boy and vice versa (...) an eight-year-old student said he has a boy’s body, dresses like a boy and walks like a boy because his mother wants to, but he says he is not a boy, but a girl (P.14, P.18, E.B)*.

In contrast, some teachers reported that transgenderity does not exist in childhood, since the child is still in the process of development: *I believe that transgenderity is something that can happen when it is occurring in adolescence, pre-adolescence, because in this phase the child is already at a pre-formed age, and then yes, they may come to decide what they want to be, but a child has no conscience to say what they want to be (P.01, P.11, E.A)*.

The second category, “The construction of gender identity and roles in childhood”, highlighted content related to the interaction mechanisms that define and demarcate gender roles in the school space. It was verified that the gender dichotomy is reinforced in the classroom from the segregation of games and toys belonging to certain genders, causing tensions and stereotyped divisions for male and female roles: *I put the girls’ and boys’ separate suggestions on the board, but he wanted makeup and as I said this present was on the girls’ side, he couldn’t ask. So, he didn’t ask for any Christmas present. He didn’t even ask for a ball, because among the boys’ suggestions there was a ball, a game, cards and, among the girls, there was makeup and a doll (...). The girl wanted to be playing more football, she was not identified so much with the doll, which was a girl thing and ended up being discriminated against by her classmates, who wouldn’t let her play (P.10, E.A; P.16, E.B)*.

The third category, “The experience of transgender children in the school context”, showed elements related to discrimination and prejudice that permeate the daily lives of transgender children: *In the classroom she suffered a lot of bullying and let herself be put down with anything, but it was because of these discoveries that she became very fragile (...) those boys who had the appearance of a girl were already criticized by their peers, called queers (...) the peers always called them gay just because they liked to use their mother’s heel (...) I have experienced in the classroom situations of boys wearing pink sandals, having grown hair, treated nails and that suffered prejudice by their peers, being called gay* (P.14, P.16, P.17, P.20, E.B).

Although they identify the tension points, the teachers are not always positioned in the face of situations of embarrassment witnessed in the classroom, the responsibility to impose themselves before their colleagues being under the discretion of the offended student: *A boy in the classroom called the classmate delicate and said that sometimes he looked like a girl. Then, this offended child imposed himself and positioned himself questioning whether this classmate was trying to refer to him as queer and said to not to call him queer, but yes, a girl* (P.02, E.A).

Another dilemma experienced in the daily life of trans students reveals the signs that “denounce them” as members of the rejected gender: *From an early age, she wears a sash on her breast and avoided using the toilets, because she did not want to go to the women’s toilet and could not use the men’s restroom, she suffered a lot from it* (P.13, E.B).

In category four, “Transgender children: How to deal with?”, the behaviors adopted by the teachers in situations involving trans children were presented. In a more pedagogical perspective, some teachers identify conflicts and seek to manage the divergences: *The times there was a conflict in the classroom because of a trans child I stopped the class for discussing on the subject matter (...). In situations of embarrassment, I always fight to combat this, I do not allow this, I talk to them trying to bring it to the most natural possible form, this depends a lot on what is going on (...)* (P.12, P.13, E.B)

On the other hand, situations were identified in which teachers are detached from the responsibility and pass it on to another professionals: *If some transgender child situation happened to me here, I would first pass the case to the social worker, so that the parents would be invited to meetings with the psychologist and go to the responsible bodies* (P.11, E.A).

Nevertheless, some teachers recognize that this should be modified; however, given the routine imposed by the institution, they end up reproducing attitudes considered exclusionary and prejudiced: *The boy wanted to ask Santa Claus for a makeup kit, but I said this present was a girl’s gift and he couldn’t ask for it. In this case I recognize that I made a mistake, I should have given him freedom to choose what he wanted (...). There was a student who this year, caught me not knowing what to do at the moment, because at the time of the presentation, which I ask to talk about where it came from and the name, I treated him as a girl, that day when the class was finished the case was forwarded to supervision* (P.04, E.A; P. 12, E.B).

In category 5, “*Discussing differences in the classroom: Is it the way?*”, the possibilities and work strategies that could contribute to the recognition of gender diversity in the school context were highlighted.

To work on the differences in the classroom, they signaled as pedagogical resources the use of contents that encourage empathy and respect to the other: *I work on empathy with the students, respect for the other, the other is who they want to be. I always work on this, but in the children’s language (...). I bring films that show a lot the issue of differences, we work on the values (...). I’ve worked with children at another school... we saw that they treated people who were transvestites with a pejorative term, and called the little schoolboy queer, so the need to talk to them about such issues was perceived. We see that only one conversation already brings greater respect to the identity of the other* (P.18, E.B; P.03, E.A; P.17, E.B).

However, they highlighted the need to insert the theme in the course plan and curriculum of the disciplines taught, in order to facilitate the approach among teachers and students: *I think that to work better on transgenderity with children it is necessary to first insert it in the curriculum, to have this theme in our course plan (...) each teacher should include this theme according to their area of activity. I am a History teacher and I can study transgender in all periods of history, but we can work in Math the number of murders of transgender people, we can work in Geography the transgender population in Brazil* (P.10, E.A; P.12, E.B).

However, some teachers do not feel prepared or do not have internal tools to address the theme: *There is a teacher here, that when it comes to the part of the book that deals with the subject of gender and sexuality, they jump and move on to the next. This has a lot to do with your religiosity, your beliefs. [They] call this filthy, dirty* (P.04, E.A).

In category 6, “Dilemmas of school and family interaction”, the limitations in the interactive processes between school and family members were emphasized. Such distancing hinders the work and makes discussions about the problems faced in their daily lives impossible. *I have tried to work with these issues, but there are situations here in the classroom that I am afraid to address, because when we approach there is trouble in for me (...) we have to approach this issue with the families, in a way that is not a confrontation for them, because they have some that see it as an affront, not as a way of trying to understand what is happening in society (...) I’ve tried to talk to the family, but often they don’t accept it and if we talk about it here, it might be the mother wanting to take her son out of school (...)* (P.06, E.A; P.15, P.16, E.B).

In addition to the fear of promoting disagreements or conflicts with the family members, some teachers highlighted the invisibility of school affairs in their children’s lives: *The parents do not participate in their child’s life at school, and we often deal with issues that I thought would create a repercussion at home and do not do so, because the parents do not seek to appropriate of what is really happening at school (...). Very often, the family does not help. We do something here at school, talk about differences, and the family undoes what was said (...) you have to prepare your child not for him to deny, but for him to prepare for the world, and only the family, along with school, can do that; the school alone is impossible* (P.03, P.06, E.A; P.13, E.B).

## Discussion

Gender identity refers to the gender with which the individuals recognize themselves socially, and can be male, female, both or neither gender. It is important to highlight that sexuality and gender identity are not the same thing, that one is not determinant in relation to the other, that is, the gender with which the person recognizes will not necessarily determine their sexuality, in the same way that the sexuality of a person will not determine their gender^(^ 23 ^)^.

Recent studies aimed at investigating the association between genotype and trans-sexuality have indicated that several genes associated with sex hormones can contribute to gender dysphoria^(^ 24 ^-^ 25 ^)^. However, it is not ruled out that the gender identity of an individual is more likely the result of a complex interaction between several genes, in addition to environmental and social factors^(^ 26 ^)^. 

On the other hand, On the other hand, an author^(^ 27 ^)^ points out that there is only transgenderity when there is the discovery of the genital organs and, from this, non-compliance with the imposed gender. In this case, in the event that a child is considered transgender, it is necessary for him/her to discover himself/herself within the body-gender process. While this does not occur in his/her world, it can be said that the attitudes and jokes he/she chooses have to do with gender roles.

A study conducted in Portugal with transgender adults, in order to analyze situations of discrimination experienced by them, verified that these adults, even children and adolescents, were faced with situations of strangeness, helplessness and violence in the school environment^(^ 28 ^-^ 30 ^)^.

In this perspective, several countries have recognized gender diversity in childhood and youth and making it increasingly visible, recognized and under-serviced by the public policies, including the educational ones. Until recently, the existing national legislation, namely the so-called “gender identity laws”, assumed that gender identification was a characteristic only of adults, creating an environment of ignorance and risk for children and their families^(^ 31 ^)^.

Attitudes such as these can be related to the attitude adopted by the teacher himself in the classroom, a fact evidenced by that although teachers identify issues involving gender and gender role in the school, through the exposure of dilemmas experienced in the daily life of transgender students, they do not position themselves in front of such situations.

It was observed in the research that classroom games do not respect the differences inherent to transgender children, which are labeled as belonging to certain genders/sexes. Games are important in childhood and have a direct relationship with the construction of gender identities. However, when conducted erroneously, they can cause early incompatibility in children between the female and male universes, besides generating disorders in the construction of their gender identity^(^ 31 ^)^.

Today, in the European context, education is one of the areas contemplated in the sense of “safeguarding the right of children and young people to education in a safe environment, free from violence, bullying, social exclusion or other forms of degrading discrimination and treatment associated with sexual orientation or gender identity”^(^ 30 ^)^.

Transphobic attitudes from educators, or even the invisibility of the topic, influenced the behavior of other students that see them as models or mentors^(^ 31 ^)^. Thus, it is clear that, despite the recognition of transphobia experienced at school, most teachers still have some resistance in addressing the topic of homosexuality and discussing gender issues, which can contribute to prejudice in their classrooms^(^ 32 ^-^ 36 ^)^. On this issue, this study showed that some teachers recognize that this must be modified; however, in view of the routine imposed by the institution, they end up reproducing attitudes considered exclusive and prejudiced^(^ 31 ^,^ 37 ^-^ 38). On the other hand, there are teachers who only reproduce their role as execution agents through the reproduction of content.

The school institution is characterized as a place that crosses the walls of learning curricular contents, as it is one of the places where the first social interactions of the individuals occur, as well as the construction of affective bonds, social identifications and especially the production of subjectivities. In this way, it is understood that the school plays a role that goes far beyond educating, and it is for this reason that it is important to discuss this very current and necessary topic, so that it will contribute with respect for diversity^(^ 31 ^,^ 39 ^)^.

Through the participants’ speeches, it is evidenced that they recognize pedagogical resources and discussions about gender identity as possibilities of work in the school context and, at the same time, signal the lack of resources, professional training and institutional support as a work limit given the theme. This is something of concern, since the silencing and/or denial of multiple sexual and gender identities are made invisible in the school universe, and they often only achieve visibility through insults and other manifestations of prejudice^(^ 40 ^)^.

mudar para “It is reported^(^ 31 ^)^ reports that the realization of respect, diversity and acceptance of trans students in the school context will only be effective given the availability of continuing education for professionals in the field of education on topics that address differences in sexual identity.

Another issue highlighted by this study, as a limit for the reception of trans children and adolescents in the school context, was religion. In this regard, it was verified that religion interferes with the way some teachers deal with gender and sexuality issues. It was observed that the religious influences and the strongest and impeding social demands of dialogs were more recurrent in School A, where the Evangelical and Catholic religions predominated.

A study carried out with people of different religions showed that, despite respecting sexual diversity, they believe and follow religious concepts and values in order to carry out their own interpretation of discordant perspectives, promoting reinterpretations that ensure a more comfortable place in the face of dissonances between institutionalized discourses and personal experiences^(^ 41 ^)^.

From the results of this study, it was also verified that, in addition to insults from classmates, transgender people are faced with other forms of violence, from the school organization itself. One of the measures that are being implemented by schools and universities is the creation of a unisex restroom, promoting non-distinction of gender, as support for transgender and non-binary people to attend public places. In addition, Resolution No. 12 of the National Council for Combating Discrimination and Promoting the Rights of Lesbians, Gays, Transvestites and Transsexuals (*Conselho Nacional de Combate à Discriminação e Promoção dos Direitos de Lésbicas, Gays, Travestis e Transexuais*, CNCD/LGBT), advises that in Brazil it is not possible to prohibit transvestites and trans-sexuals from using restrooms according to their gender identity^(^ 42 ^)^.

However, it is verified that physical and psychological violence has been affecting trans people in different settings, from their exposure to vexing and disrespectful situations in their own family and school environment^(^ 43 ^)^. As a result, the number of mental illness and suicide cases among these people is significant. In the year 2016 alone, *Rede Trans Brasil* managed to catalog 12 suicide cases of trans people^(^ 44 ^)^, with the majority being among young people between 15 and 29 years old, especially women. Recently, the Trans-sexualities and Public Health in Brazil report, from the Center for Human Rights and LGBT Citizenship and the Anthropology and Archeology Department of the Federal University of Minas Gerais (*Universidade Federal de Minas Gerais*, UFMG), pointed out that 85.7% of the trans men have already thought about suicide or attempted it at some point in their lives^(^ 45 ^)^.

A study carried out by the Williams Institute (UCLA) showed a high prevalence of suicide among trans men (46%), especially between 18 and 24 years old (45%). Among the main motivations for attempting suicide, in addition to mental condition, experiences of persecution, harassment, violence, discrimination and rejection were highlighted, factors that together lead the individual to a state of greater vulnerability^(^ 46 ^)^.

However, it is clear that transgender person do not escape the school environment; they are in fact aggressively and impartially expelled. Therefore, they are at the mercy of a society that is unlikely to point out any other way than prostitution, leading them to a life without social, labor and personal security guarantees^(^ 47 ^)^.

To deal with this, it would be necessary to create public education policies aimed at social transformation, with the induction of teacher training projects, establishment of awareness policies and the creation of school discussion groups on gender issues^(^ 47 ^)^. The school is considered as one of the main ways for facing discrimination and prejudice among trans people. Therefore, it is necessary to reflect on the relationships and rights of all individuals. It is only possible to transform something that is known. It is therefore essential to expand spaces for reflection and access to information so that human rights are accessible to all, based on respect, acceptance, dialog and co-living with the differences.

In relation to the limits of the study, it is emphasized that the data presented reflect the reality of two schools in a capital of northeastern Brazil, of only one city, which reflects specific sociocultural conditions. However, considering the potential of qualitative data analysis and the scarcity of studies on childhood transgenderity, this study contributes to the advancement of scientific knowledge and to Nursing, by revealing information that may facilitate the development of educational public policies and strategies for action, with the purpose of guaranteeing welcoming and respect for transgender children in the schools. In addition, the study can bring contributions to the discussion of a current issue, but still invisible to the eyes of the school and society.

## Conclusion

This research was based on the social need to propitiate visibility to transgender people, expanding the possibilities of discussion favoring the guarantees of rights aimed at education, health and citizenship.

The investigation of the teachers’ conceptions of transgenderity in childhood provided opportunities for several reflections on the possibilities/limits of working with transgender children in the school context. Childhood transgenderity is an unquestionable reality; however, despite increasing advances in the political and social spheres, the schools have difficulties in promoting the inclusion of trans children. Schools A and B have different social, religious, cultural and personal positions, which differentiate them in the way they are conducting diversities. While at School A a more conservative and heteronormative stance predominates, at School B the approaches are more open, participatory and welcoming.

However, regardless of the positions of the Schools, it was observed that the dilemmas are the same, possibly permeated by the deficient appropriation and interest of the families in the educational process of their sons and daughters. It becomes necessary to create effective strategies aimed at raising parents’ awareness, in addition to promoting open spaces for dialog between teachers, family members and other social support networks on issues involving children and adolescents.

Moreover, it is necessary to develop pedagogical practices that reflect and discuss issues about the constitution of the subjects in the school context, so that in fact there is promotion of the inclusion of trans children, contributing to the construction of their citizenship and the effectiveness of one of the basic rights settled by the constitution: education.

In the field of health, especially in Nursing, the importance of the study is grounded since, as a professional with a historical approach to the health of the student, the nurse has the role of appropriating the necessary knowledge for the provision of care, reinforcing and encouraging inclusive attitudes and planning intervention strategies aimed at different social groups.
